# Amantadine Toxicity Mimicking Holmes Tremor Following Diuretic Dose Escalation: A Case Report

**DOI:** 10.7759/cureus.101555

**Published:** 2026-01-14

**Authors:** Akio Nakashima, Motoyasu Miyazaki, Katsuko Tachikawa, Osamu Imakyure

**Affiliations:** 1 Department of Pharmacy, Fukuoka University Chikushi Hospital, Chikushino, JPN; 2 Department of Hospital Pharmacy, Faculty of Pharmaceutical Sciences, Fukuoka University, Fukuoka, JPN; 3 Department of Gastroenterology, Fukuoka University Chikushi Hospital, Chikushino, JPN

**Keywords:** acute renal impairment, amantadine, diuretic, holmes tremor, toxic accumulation

## Abstract

A 69-year-old man with several comorbidities, including hypertension, diabetes mellitus, sequelae of cerebral infarction, and alcoholic liver disease, was admitted to the hospital for transcatheter arterial chemoembolization to treat recurrent hepatocellular carcinoma. Amantadine is eliminated unchanged by the kidneys; even standard therapeutic doses may lead to toxic accumulation when renal function deteriorates. As amantadine neurotoxicity can present with tremor phenotypes resembling cerebellar or Holmes-like tremor, diagnosis is often challenging. In this case, the spironolactone dose was doubled three months before admission, followed by a marked decline in renal function. Two months before hospitalization, the patient developed progressive tremor, gait instability, and dysarthria, and was initially diagnosed with Holmes tremor based on the neurological findings. Although levodopa therapy was initiated, a pharmacist recommended measuring plasma amantadine concentration in light of recent renal impairment. Despite blood sampling 35 hours after the last dose, the amantadine concentration was markedly elevated at 3,935 ng/mL, far exceeding known toxic thresholds. After amantadine discontinuation, plasma concentrations declined, and neurological symptoms gradually improved, with approximately 80% resolution by hospital day nine and complete resolution by six months. Renal function also recovered during follow-up without recurrence of tremor. This case suggests that measuring plasma amantadine concentration should be considered when patients receiving amantadine develop new tremor or gait disturbance in cases with renal dysfunction, while acknowledging the diagnostic uncertainty inherent in single-case observations.

## Introduction

Amantadine is widely used in neurological practice, including for Parkinson’s disease-related dyskinesia, post-stroke apathy, and other conditions associated with reduced motivation or motor activity. As amantadine is excreted unchanged via the kidneys, impaired renal function can substantially prolong its elimination half-life, resulting in drug accumulation even at standard doses [1,2]. Reported manifestations of amantadine neurotoxicity include delirium, hallucinations, myoclonus, and various tremor phenotypes [3-5].

Holmes tremor is a symptomatic tremor characterized by a combination of rest, postural, and kinetic components, typically involving proximal and distal muscles. It is classically associated with lesions of the cerebellothalamic and nigrostriatal pathways, but its clinical presentation can overlap with other movement disorders [6-8]. Importantly, renal dysfunction itself may also contribute to neurological symptoms, further complicating diagnostic interpretation.

Recent literature suggests that Holmes tremor does not arise from a single anatomical or neurochemical abnormality but rather from heterogeneous mechanisms involving the disruption of cerebellothalamic and dopaminergic pathways [9]. Consequently, drug-induced movement disorders may closely resemble Holmes tremor, leading to diagnostic challenges. Here, we report a case in which severe amantadine accumulation secondary to diuretic-associated renal impairment produced a Holmes-like tremor phenotype. This case highlights the diagnostic challenge and emphasizes the clinical value of measuring plasma amantadine concentration, particularly when renal function acutely declines.

## Case presentation

A 69-year-old man suffering from hypertension, diabetes mellitus, sequelae of cerebral infarction, alcoholic liver cirrhosis, hepatocellular carcinoma, and angina pectoris was admitted to the hospital for transcatheter arterial chemoembolization (TACE) to treat recurrent hepatocellular carcinoma. Compared with laboratory data from three months prior, his renal function had declined by the time of admission (Table 1), so TACE was postponed.

**Table 1:** Major laboratory values three months before admission and at the time of admission. WBC = white blood cells; AST = aspartate aminotransferase; ALT = alanine aminotransferase; LDH = lactate dehydrogenase; ALP = alkaline phosphatase; γ-GTP = γ-glutamyl transpeptidase; CK = creatine kinase; BUN = blood urea nitrogen; eGFR = estimated glomerular filtration rate

Parameter	3 months before admission	At admission	Unit
WBC	3,400	3,700	/μL
Hemoglobin	8.9	11.5	g/dL
Platelet count	7.4	5.3	×10⁴/μL
Total protein	6.1	6.2	g/dL
Albumin	2.3	3.3	g/dL
Total bilirubin	1.3	2.2	mg/dL
AST	39	30	IU/L
ALT	20	27	IU/L
LDH	311	226	IU/L
ALP	468	444	IU/L
γ-GTP	156	229	IU/L
Blood ammonia	–	117	μg/dL
CK	180	135	IU/L
BUN	13	40	mg/dL
Creatinine	1.47	4.18	mg/dL
eGFR	37.9	12.0	mL/minute/1.73 m²
Sodium	145	140	mEq/L
Potassium	4.1	4.7	mEq/L

During the initial pharmacist interview, the patient reported, “I started having tremors two months ago. One month ago, the tremor worsened so much that I couldn’t eat well. I used to walk with a cane even though my right leg was weak from an old stroke, but the tremor became so severe that I couldn’t use the cane anymore.” In his medication notebook (Table 2), the pharmacist observed that the spironolactone dose had been doubled approximately three months before, from 25 mg/day to 50 mg/day, raising suspicion of dehydration-induced renal impairment. The patient had been receiving amantadine hydrochloride 100 mg/day for several years to treat reduced motivation and apathy associated with post-stroke sequelae, not for Parkinson’s disease or parkinsonian symptoms. He tolerated amantadine without adverse effects before this admission. Because the patient took amantadine without issues before the diuretic dose escalation, amantadine accumulation due to reduced renal clearance was strongly suspected.

**Table 2:** Medication list at the time of admission.

Medication	Daily dose
Amantadine hydrochloride	100 mg
Clopidogrel sulfate	75 mg
Lansoprazole	30 mg
Bisoprolol fumarate	2.5 mg
Sitagliptin phosphate hydrate	12.5 mg
Zolpidem tartrate	10 mg
Flunitrazepam	1 mg
Tamsulosin hydrochloride	0.4 mg
Furosemide	20 mg
Spironolactone	50 mg
Branched-chain amino acids (isoleucine, leucine, valine)	14.22 g
Sodium ferrous citrate	100 mg
Levocetirizine hydrochloride	5 mg

**Figure 1:**
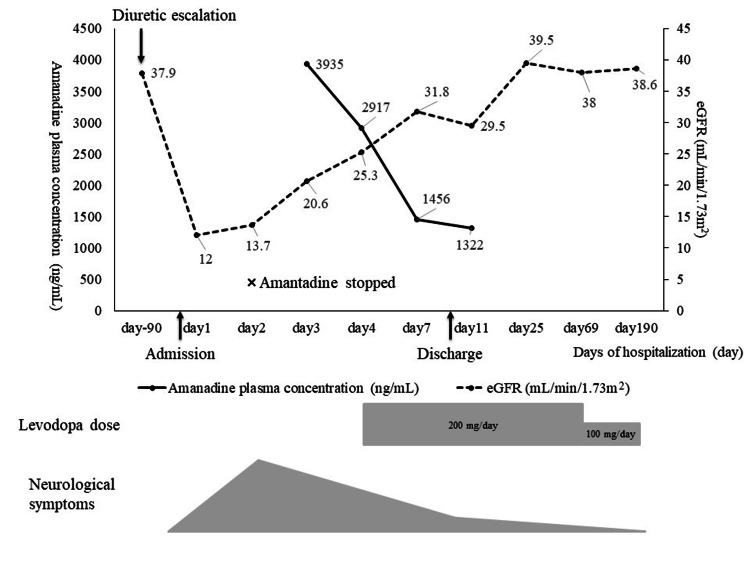
Clinical timeline of the patient’s course. eGFR = estimated glomerular filtration rate

The pharmacist recommended immediate discontinuation of amantadine (hospital day two) and requested that the plasma amantadine concentration be measured. All oral medications, including amantadine, were discontinued on hospital day two, and treatment for acute renal failure was initiated. On hospital day four, a neurology consultation was requested due to involuntary upper-limb movements and gait instability. The patient was alert and fully aware of the time, place, and his personal information. An ocular examination showed saccadic intrusions. No facial palsy was observed. Voice tremor and mild dysarthria were seen, although the patient retained the ability to swallow. Tremor was present when the upper limbs were elevated and during standing. Manual Muscle Testing revealed grade 4 strength in all extremities, and residual right-sided hemiparesis from a previous cerebral infarction was evident. Based on the neurological findings of postural and kinetic tremor worsening upon movement initiation, the neurologist diagnosed Holmes tremor originating from the brainstem or cerebellum. Alcohol-related cerebellar dysfunction and sequelae of stroke were considered possible differential diagnoses. A head CT revealed no acute lesions. Levodopa/carbidopa (200 mg/day) was initiated according to the neurologist’s recommendation. Because the assay was outsourced, the amantadine plasma concentration results were available only after patient discharge. Despite blood sampling 35 hours after the last dose, the plasma amantadine level was highly elevated at 3,935 ng/mL, far exceeding toxic levels. As shown in Figure 1, which presents the detailed clinical timeline, amantadine concentrations gradually declined following discontinuation, accompanied by progressive improvement in neurological symptoms. He was discharged on hospital day nine (eight days after amantadine discontinuation), by which time approximately 80% of the tremor had resolved. Two months after discharge, the levodopa dose was reduced to 100 mg/day due to further symptom improvement, and levodopa was discontinued entirely six months after discharge. At that time, the tremor had fully resolved. Renal function also improved during follow-up, with estimated glomerular filtration rate values of 38.0 mL/min/1.73 m² at two months and 38.6 mL/min/1.73 m² at six months after discharge, without the recurrence of tremor or other involuntary movements.

## Discussion

This case illustrates the diagnostic complexity that can arise when amantadine toxicity presents with tremor phenotypes resembling Holmes tremor. Two clinically relevant considerations emerge from this case. First, escalating the diuretic dose may precipitate amantadine accumulation by inducing acute renal impairment. Second, amantadine-related movement disorders can closely mimic cerebellar or brainstem-origin tremors, potentially leading to diagnostic misclassification.

When tremor and myoclonus coexist as manifestations of amantadine neurotoxicity, the resulting phenotype may closely resemble cerebellar or Holmes tremor. This clinical overlap has been described in previous reports of amantadine-induced movement disorders, in which abnormal involuntary movements mimicked structural or degenerative neurological conditions [3,5]. This overlap can contribute to diagnostic misclassification, especially in patients with preexisting neurological comorbidities.

Holmes tremor is attributed to lesions affecting multiple neural pathways, including the dopaminergic nigrostriatal system and cerebello-thalamo-cortical or dentato-rubro-thalamic circuits. Disruption of these pathways has been demonstrated in individual case reports of dopaminergic dysfunction and in broader reviews of Holmes tremor pathophysiology [9-11]. Lesions affecting these interconnected pathways may produce mixed tremor phenotypes that include resting, postural, and kinetic components. Pharmacological perturbation of these circuits, such as excessive dopaminergic or N-methyl-D-aspartate (NMDA)-related modulation, may theoretically produce a Holmes tremor-like presentation without new structural lesions.

From a pharmacological perspective, amantadine acts on the central nervous system by enhancing dopaminergic neurotransmission and antagonizing NMDA receptors [9,10]. These mechanisms influence cerebellothalamic and basal ganglia circuits involved in motor control. Excessive dopaminergic activity and altered glutamatergic signaling due to drug accumulation may therefore contribute to the emergence of tremor and myoclonus, although a direct causal relationship cannot be established from a single case.

In this patient, spironolactone dose escalation likely contributed to dehydration and renal impairment, reducing amantadine clearance and precipitating toxic accumulation. Importantly, the patient had been receiving amantadine for post-stroke apathy rather than Parkinson’s disease, and the drug was well-tolerated for several years before the acute decline in renal function. As amantadine is eliminated unchanged by renal excretion, deterioration in renal function markedly prolongs its elimination half-life and increases systemic exposure. Previous pharmacokinetic studies have demonstrated that renal impairment substantially alters amantadine clearance, predisposing patients to neurotoxicity [1,2]. In this context, acute renal impairment following diuretic dose escalation may be a critical risk factor for developing amantadine-induced movement disorders.

Although levodopa was initiated after the neurological diagnosis, symptom improvement temporally coincided with amantadine discontinuation and declining plasma concentrations, as illustrated in Figure 1. As levodopa was administered during the recovery phase, the relative contribution of each intervention cannot be determined with certainty. Nevertheless, the absence of symptom recurrence despite the recovery of renal function and subsequent discontinuation of levodopa supports the interpretation that amantadine accumulation played a substantial role in symptom development.

Plasma amantadine concentration is not routinely measured because assays are not widely available and results are often delayed. However, this case demonstrates that measurement can be critical in certain patients, particularly when new neurological symptoms arise in cases with renal dysfunction or recent medication changes. The retrospective confirmation of toxicity is a limitation of this report, and causality cannot be definitively established from a single case. Nonetheless, the temporal association among renal impairment, drug accumulation, and symptom resolution provides clinically meaningful insights.

## Conclusions

Amantadine toxicity may occur even at standard doses when renal function deteriorates, including after diuretic dose escalation. Because the resulting movement disorder may closely resemble Holmes-like tremor, differentiation based solely on neurological findings can be difficult. This case suggests that measuring plasma amantadine concentration should be considered when patients receiving amantadine develop new tremor or gait disturbance in cases with renal dysfunction, while acknowledging the diagnostic uncertainty inherent in single-case observations.
